# The evolutionary cost of homophily: Social stratification facilitates stable variant coexistence and increased rates of evolution in host-associated pathogens

**DOI:** 10.1371/journal.pcbi.1012619

**Published:** 2024-11-22

**Authors:** Shuanger Li, Davorka Gulisija, Oana Carja

**Affiliations:** 1 Computational Biology Department, School of Computer Science, Carnegie Mellon University, Pittsburgh, Pennsylvania, United States of America; 2 Department of Biology, University of New Mexico, Albuquerque, New Mexico, United States of America; University of Zurich, SWITZERLAND

## Abstract

Coexistence of multiple strains of a pathogen in a host population can present significant challenges to vaccine development or treatment efficacy. Here we discuss a novel mechanism that can increase rates of long-lived strain polymorphism, rooted in the presence of social structure in a host population. We show that social preference of interaction, in conjunction with differences in immunity between host subgroups, can exert varying selection pressure on pathogen strains, creating a balancing mechanism that supports stable viral coexistence, independent of other known mechanisms. We use population genetic models to study rates of pathogen heterozygosity as a function of population size, host population composition, mutant strain fitness differences and host social preferences of interaction. We also show that even small periodic epochs of host population stratification can lead to elevated strain coexistence. These results are robust to varying social preferences of interaction, overall differences in strain fitnesses, and spatial heterogeneity in host population composition. Our results highlight the role of host population social stratification in increasing rates of pathogen strain diversity, with effects that should be considered when designing policies or treatments with a long-term view of curbing pathogen evolution.

## Author summary

Understanding the mechanisms that shape and protect the maintenance of diversity in a pathogen population is of significant importance to curbing their spread in the population. Here we discuss a new diversity-promoting mechanism where, unlike typical balancing mechanisms, the diversity is not buffered from selection in protected life-stages of the pathogen or distinct spatially separated subpopulations, but instead, variant diversity is protected by the social interaction structure of the host population. In turn, increased diversity in the pathogen population increases the rate of their evolution, incurring the health cost to the host population, which we refer to as the evolutionary cost of homophily. Our model can also be interpreted through the lens of contagious social behaviors and showcases how the social architecture of the population can shape the coexistence of a diverse array of cultural variants. For both cultural or biological pathogens, understanding the evolutionary mechanisms that contribute to transient or stable variant diversity is essential for designing responses and policies that prevent increases in strain repertoire of pathogenic variants in the population.

## Introduction

From genes to communities, natural systems are characterized by high rates of variant coexistence and understanding the mechanisms that shape and protect the maintenance of diversity in a population is a central problem in biology. Evolutionary mechanisms that are known to promote long-lived, non-neutral strain polymorphism are usually balancing mechanisms, arising from direct negative frequency dependence [1] or from spatially heterogeneous selection pressure [2]. An additional class of mechanisms that have been theoretically shown to promote polymorphism across a wide range of evolutionary scenarios are storage effects, initially recognized in studies of species coexistence [3–6]. Storage effects can promote coexistence when there exist patches of habitat (or specific life stages, or genetic backgrounds) where selection against deleterious variants is diminished, compared to other patches [5, 7–10]. As a result, this effect can store long-lived polymorphism in systems with spatial, life-stage or genetic heterogeneity [7, 8, 11–13].

Here we ask whether patterns of preferential social interaction between individuals can, similarly, generate enough differential in selection pressure to stably increase rates of variant coexistence in a population. Biased preferences of interaction and their effects on population segregation have been widely documented in the social science literature, starting with the ground-breaking studies of Thomas C. Schelling on how micro-motives can shape macro-patterns of population behavior [14–16]. Across many dimensions of phenotypes (including physical, cultural, and attitudinal characteristics), humans exhibit high levels of homophily, the tendency to interact with others of similar type, in social tie formation and patterns of social interaction [17–21, 53]. Moreover, homophily has been shown to significantly shape patterns of pathogen or cultural variant spread. For example, the structure of sexual networks of interaction has been shown to impact the spread of HIV [22] and opinion diffusion through social networks has been shown to influence individuals’ decision for vaccination, thus indirectly also shaping contact-dependent vulnerability to pathogens [23]. Recent studies have also investigated the impact of homophily on infection dynamics during the COVID-19 pandemic [24, 25], and highlighted the importance of considering homophily when developing vaccination strategies [26, 27].

In parallel, empirical work has explored how denser patterns of interaction can lead to higher levels of phenotypic and/or immunological similarity between individuals. Local environmental conditions determined by social interactions have been proposed as key determinants of human cellular immune systems, creating different patterns of interaction between different immunological profiles that could significantly impact pathogen spread and diversity [28, 29]. Vulnerabilities to infections correlate with factors like age, ethnicity, exposure histories, and immune system competence [30–38]. Similarly, extensive bacterial strain sharing across human populations, with distinct mother-to-infant, intra-household and intra-population transmission patterns, points to the important role of the contact network in shaping microbiome diversity and transmission [39–41].

The influence of social patterns of preferential interaction between population groups that are more immunologically and phenotypically similar on the long-term evolutionary dynamics of the pathogen population remains unclear. Here we use a population genetic model to study how social biases of interaction between host population subgroups with varying immune responses or phenotypic characteristics shape rates of pathogen strain polymorphism and long-lived coexistence. We show that, as strains of varying virulence enter the host population, these potential differences in immunity between host subgroups of interaction can promote polymorphism-protecting heterogeneous selective pressure against the pathogen. Therefore, the prevalence of a strain will be increased when circulating within the host group favoring the strain and will be constrained within the social group where the strain is disadvantaged.

This dynamic generates a diversity-promoting mechanism where, unlike typical balancing mechanisms, the diversity is not stored (buffered from selection) in protected life-stages of the pathogen or distinct spatially separated subpopulations, but instead variant diversity is protected by the social interaction structure of the host population. In turn, increased diversity in the pathogen population increases the rate of their evolution, incurring the health cost to the host population, which we refer to as the evolutionary cost of homophily. We show that increased rates of homophily in the host population promote increased rates of heterozygosity and long-lived multi-strain coexistence and study the role of temporal variance in the host social preference of interaction. We find that periodic amplification of existing homophily in the host population, even when short-term, can significantly increase rates of pathogen diversity and polymorphism.

Our model can also be interpreted through the lens of contagious social behaviors, where two cultural variants are maintained in the population due to the preferential interaction between individuals with similar phenotypic characteristics, such as conformist or anti-conformist behaviors [42, 43]. For both cultural or biological pathogens, understanding the evolutionary mechanisms that contribute to transient or stable variant diversity is essential for designing responses and policies that prevent increases in strain repertoire of pathogenic variants in the population.

## Model description

To examine the effect of social preferences of interaction on rates of host-associated pathogen diversity and polymorphism, we use a Wright-Fisher model to describe changes in strain frequencies within a host population of fixed size *N*. The host population consists of two different types of individuals, with the different host immune-phenotypes denoted by *S* and *A*, interpreted here as individuals with different sensitivity to pathogen virulence, and broadly referred to as symptomatic (*S*) and asymptomatic phenotypes (*A*). These host phenotypic differences correspond to potential differences in immune system composition and effectiveness between people with different genetic backgrounds or in different age groups, which can affect host symptoms and disease progression when infected [30–38]. Since the host-population generation times correspond to an equivalent of many microbial generations, we assume that the proportion of *A* to *S* individuals in the host population does not change through the time of pathogen evolutionary dynamics.

**Fig 1:**
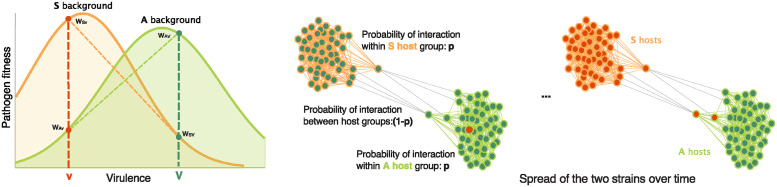
Illustration of the model. Pathogen fitness is determined by the interplay between the virulence of the strain and the immune vulnerability of the host it infects. Left: The more virulent strain *V* (dark green) is assumed to have higher fitness on the A-type (light green) hosts (*w*_*AV*_ > *w*_*Av*_), while strain *v* (dark orange) has higher fitness on the S-type (light orange) hosts (*w*_*Sv*_ > *w*_*SV*_). Middle and right: Illustration of host social stratification and implications for pathogen coexistence, over time. Host genotypes, *A* (light green) and *S* (light orange), are indicated by the bounding circles, while the pathogen genotypes they carry, *v* (dark green) and *V* (dark orange), are indicated by the color fill. The host population is socially structured, with *A* and *S* individuals more likely to interact with other individuals of the same phenotype. At the beginning of the simulation, *v* is the resident strain, and a new mutant *V* enters the population. Through time, the strains may concentrate on their preferred hosts.

Each host individual carries one (and only one) of two different pathogen strains, denoted here by *v*/*V*. We assume that these two strains differ in their host-pathogen affinity. For example, two different viral strains could differ in their virulence, with the *v* strain having a lower virulence than the *V* strain. Pathogen fitness is determined by the interplay between the virulence of the strain and the immune vulnerability of the host it infects [44]. We model a scenario with trade-off viral fitness distributions on the two host backgrounds *A* and *S*, with optimal virulence smaller on the more sensitive, symptomatic *S* background than on the asymptomatic *A* background (Fig 1). It assumes that the benefits of a higher transmission rate can only accrue if the host is healthy enough to interact with other hosts and transmit the strain further. Pathogens with the highest fitness are those with an intermediate level of virulence, striking a balance between within-host production of more transmission forms per unit time and infection strength and length.

This model can also be applied to understand the evolutionary dynamics of two ‘contagious’ cultural traits (for example risk aversion or risk taking behaviors that affect the health of the individual), with different levels of expression on two different individual backgrounds (for example, conformist and anti-conformist hosts or hosts of different status or cultural background [42, 43]), where trait fitness and transmissibility to other hosts depends on the virulence of the trait, in interplay with its host phenotype and sensitivity to the cultural behavior.

We use the following fitness scheme:

$$\begin{array}{lll} & \text{Strain}\;V & \text{Strain}\;v\\ \text{Fitness}\;\text{on}\;\text{host}\;A\;\text{background} & w_{AV}=1+s_{1} & w_{Av}=1\\ \text{Fitness}\;\text{on}\;\text{host}\;S\;\text{background} & w_{SV}=1 & w_{Sv}=1+s_{2}. \end{array}$$

The more virulent strain *V* is assumed to have higher fitness on the *A*-type hosts (determined by positive selection coefficient *s*_1_), while strain *v* has higher fitness on the *S*-type hosts (determined by positive selection coefficient *s*_2_) (Fig 1). For example, one can interpret the model as symptomatic hosts *S*, infected with a very virulent strain being likely to show symptoms early and reduce social activities, effectively leading to a reduced transmission rate and a selective disadvantage of the *V* strain on this background. On the other hand, immunocompetent hosts *A* show fewer symptoms, even with the *V* strain, which can contribute to higher host transmission rates, thus conferring a selective advantage to the *V* strain on this host background, as has been described in models of viral-host dynamics [45]. Similarly, for the *v* strain, the lower virulence allows for higher transmission on the symptomatic *S* background. Trade-offs in virulence, transmissibility and pathogen fitness [46–50] are complex and can depend on many parameters. However, the fitness scheme we use is a good baseline model, elegant in its simplicity, to study how the composition and interaction patterns in the host population affect pathogen rates of coexistence.

Since our goal is to study the evolution of the pathogen in the population of infected individuals, we assume that all hosts in the population are infected with exactly one of the two strains, the probability of coinfection is considered negligible and, initially, at time *t* = 0, all hosts are assumed to be infected with the wild-type strain *v*. In the first generation of each simulation run, a new mutation introduces the more virulent strain *V* in one of the hosts. At each generation, each original host can transmit its pathogen to new hosts, with probability determined by the pathogen strain fitness. Hence, the strain frequencies of the next generation are generated by sampling with replacement proportional to pathogen strain fitnesses and host interaction patterns and we study the change in frequencies of the two pathogen strains in the population. We do not allow for recurrent mutation.

An important aspect of the model is that hosts do not interact randomly, but instead, preferentially with other hosts belonging to the same social strata (henceforth group). That is, we assume that each host belongs to one of the two groups and will interact with a random individual from their own group with probability *p*, a social preference parameter. In our model, social preference of interaction does not depend on group sizes. This is due to the fact that interactions are probability-based: an individual will get infected with a probability *p* by someone from their group and (1 − *p*) by someone from another group (Fig 1). Therefore, if the two social strata are of different sizes, individuals from a smaller group infect more individuals from the larger group and vice versa, which would happen, for example, when an “outsider” individual interacts or moves through a different, more densely populated segment of the population.

In addition, when groups are phenotypically homogenous, the social preference of interaction, or homophily, does not depend on the proportion of the different phenotypes in the population. Note that when the two groups are of equal sizes, a population with randomly interacting individuals would have *p* = 0.5. The setup above directly models the possibility of higher viral transmission opportunities within groups than between groups [15, 16, 19, 51, 52]. In what follows, we make a simplifying assumption that *p* is equal for the two groups. Most of our simulations also assume that groups are large and phenotypically homogenous (*A* or *S*). Thus, *p* becomes the probability that an individual will interact with an individual of the same phenotype at the population level. In extensions of the model where groups are phenotypically heterogenous (a mixture of *A* and *S*), the probability of transmission to a host of the same phenotype in the same group becomes a function of *p* and the proportion of the phenotype within the group.

Strain coexistence and stable polymorphism in the host population can be quantified by the cumulative expected heterozygosity

$$\begin{array}{c} H=2E(\sum_{t=1}^{t=T}q_{t}\left(1-q_{t}\right)), \end{array}$$ (1)

where *q*_*t*_ represents the frequency of strain *V* at time *t* and the expectation is over independent simulations runs (ending at times *T*) [54]. Here *H* represents the expected sum of heterozygosities over the lifetime of a novel mutant and it can quantify departure from neutrality, independent of population size. When selection is neutral and allele or strain frequencies are only affected by genetic drift, under a randomly mating population, the cumulative expected heterozygosity *H*_*neutral*_ has been shown to be equal to two, regardless of the population size [54], and thus *H* > 2 describes an elevated level of polymorphism (i.e. coexistence) relative to that under neutrality. It can also be shown that the expected heterozygosity in a haploid population under recurrent mutation *μ* equals *HNμ* [54].

Social preference of interaction can however generate population subdivision and inflate the cumulative expected heterozygosity *H*. We correct for the effects of population stratification by measuring the subdivided cumulative expected heterozygosity,

$$\begin{array}{c} H_{L}=2E(\sum_{t}\sum_{i}\frac{q_{i,t}\left(1-q_{i,t}\right)N_{i}}{N}), \end{array}$$ (2)

where *q*_*i*,*t*_ represents the frequency of strain *V* in host social interaction group *i* at time *t*, and *N*_*i*_ represents the size of the host population of immuno-phenotype *i*. The subdivided cumulative expected heterozygosity *H*_*L*_ has also been shown to equal two under neutrality, irrespective of the specifics of population subdivision [9]. Therefore, when the levels of polymorphism in the population exceed those under drift, we expect *H*_*L*_ > 2 i.e., we observe strain coexistence beyond that expected by chance.

We study the subdivided cumulative expected heterozygosity *H*_*L*_ as a function of the social preference of interaction *p*, the selection coefficients *s*_1_ and *s*_2_, the population size *N*, and the ratio of the two host phenotypes frequencies in the population.

We also extend this baseline model to show its robustness to various relaxations of our assumptions:

**Temporally varying preference of social interaction**. Social preferences of interaction are known to temporally vary [55]. To relax our requirement of constant social interaction preferences, we consider a model in which the hosts change their interaction preferences periodically. We assume hosts interact with preference *p* for *n*_1_ virus generations, and interact without preference for *n*_2_ virus generations. Here, *n*_1_ and *n*_2_ need not be equal.

**Variance in immune phenotypes within host groups or social strata**. To reflect the natural demographic variance in host immuno-phenotype and test the model robustness to variance in immune phenotypes within host subgroups of preferential social interaction, we develop an extension of the model in which we model two randomly interacting population subgroups of fixed population sizes $\frac{1}{2}N$, each containing a mixture of *A* and *S* individuals. Therefore, the host phenotype composition is different between the two host subgroups and the probability of interaction and pathogen transmission is now dependent on the *A* and *S* phenotype abundances in each subpopulation (Fig A in S1 Text).

**Changes in host population size and periodic host bottlenecks**. Initially, we assume that the infection rates in the host population are such to generate a large number of infected hosts proxied by a constant population size *N*. To accommodate for seasonal changes in infection rates, we also model variable *N*, where the infected population size is initiated at a point in a period of oscillating sizes ranging from 0.05*N* to *N* to 0.05*N*, repeatedly. This generates periods of strong bottlenecks in the affected population.

### Implementation and simulation details

We use Monte Carlo simulations [56] to compute the subdivided cumulative expected heterozygosity *H*_*L*_ using an ensemble of at least 5 × 10^6^ independent replicate populations of size *N* = 10^5^. In addition to the subdivided cumulative diversity, we record the proportion of simulation runs in which long-lived strain polymorphism is maintained in the population at 100*N* generations. We also record the time of fixation or loss of the two strains, which allows us to quantify the duration of protected polymorphism that perishes. Elevated *H*_*L*_ is compared with values from control simulations either under strain neutrality (all fitness coefficients equal to one) or under a scenario of immune reaction homogeneity across the population.

Simulations are terminated when the new mutant *V* fixes or goes extinct in the population, or, in the case of stable long-lived polymorphism, the two strains coexist over a range of 10^7^ (or 100*N*) generations. We assume a host can only be infected by a pathogen strain obtained from one other host at each generation, there is no co-infection, and the effects of lethality or immunity between generations are negligible.

Unless otherwise specified, the population contains equal proportions of *A* and *S* hosts. In the case of temporally varying preference of social interaction, we implement periodic changes in the homophily parameter *p* at deterministic time periods of *n*_1_ and *n*_2_ generations, repeatedly. We implement both symmetric, as well as asymmetric temporal periods, where the duration of the time interval with social preference of interaction can be different from the duration of the time intervals of random interaction and mixing between the hosts.

For the extension of the model to incorporate immune-heterogenous host subgroups, we simulate *R* = 10^7^ independent replicate populations of size *N* = 10^5^ (each host group is assumed to have *N* = 50000). We assume two different social subgroups or social strata, each containing a mixture of *A* and *S* hosts, with host phenotype composition (i.e. proportions of *A* and *S* hosts) different between the two subgroups. In this version of the model, we assume that hosts interact with social preference *p* with other hosts in their social strata (regardless of host phenotype) and, with probability of interaction (1 − *p*) with hosts in the other social subgroup (Fig A in S1 Text). This application could represent different schools, for example, where individuals of different ages are more likely to interact within their school or social strata than between schools, regardless of their immuno-phenotype. Transmission here is now dependent on the *A* and *S* abundances in the two population subgroups.

## Results

### Social preferences of interaction promote long-lived pathogen coexistence

**Fig 2:**
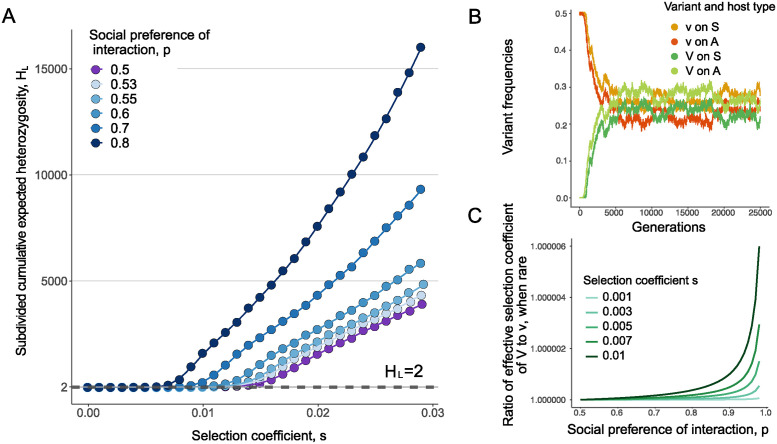
Social preference of interaction promotes increased pathogen strain coexistence. (**A**) The subdivided cumulative expected heterozygosity, *H*_*L*_ in a population of size *N* = 10^5^ as a function of the selection coefficient *s*_1_ = *s*_2_ = *s*. Here, the social preference of interaction *p* is as presented in the legend. The dots represent ensemble averages across 10^7^ replicate Monte Carlo simulations, while the lines represent cubic spline regression. The dotted straight line shows *H*_*neutral*_ = 2. (**B**) Population frequencies of each strain-host combination through time for one run of the simulation, with *N* = 10^5^, *p* = 0.95, and *s*_1_ = *s*_2_ = 0.02 and equal frequencies of *A* and *S* individuals. (**C**) Theoretically-derived relative effective selection difference between the two strains when *V* is rare in the population, as a function of the strength of selection and social preference. This effective selection difference increases with both the strength of selection *s* and the social preference of interaction *p*, showcasing the role of negative frequency dependence in shaping patterns of heterozygosity and polymorphism.

Even in a randomly interacting population, the subdivided cumulative expected heterozygosity *H*_*L*_ is a magnitude higher than drift controls for relatively low values of the selective differential *s* and further increases with increasing selective differential *s* (Fig 2A and Fig B in S1 Text). This is because even though the individuals in the population are randomly interacting, the two host immuno-phenotypes in effect create heterogenous selection, with each host immune-type providing opposite selection pressure on the virus from the other. This protects pathogen diversity in the population for longer than expected under controls.

As the social stratification of the host population increases (*p* > 0.5), rates of strain heterozygosity raise rapidly, even for small values of social bias of interaction *p* (Fig 2A and Fig C in S1 Text). This rise in levels of cumulative subdivided heterozygosity *H*_*L*_ is more pronounced as the selective differentials between the two fitness strains become stronger. For large enough selection and social preference of interaction, we observe a long-lived polymorphism that stably persists for at least 100*N* generations under a large set of parameter combinations. We sample frequency trajectories to gain insight into the relative evolutionary dynamics between the two strains and find that their frequencies, *f*_*V*_ and *f*_*v*_, oscillate inside a small stable frequency range where *V* preferentially infects *A* hosts and *v* preferentially infects *S* hosts (Fig 2B) and this coexistence of *v* and *V* can be maintained for extended periods of time (Fig D in S1 Text).

Interestingly, even though strains are each preferred on a single host immune type and with strong social stratification (*p* = 0.95), we observe high frequencies of the two strains on both *A* and *S* individuals. In other words, *V* strain proliferation on their preferred *A* hosts, also drive high rates of *V* on *S* hosts in a relatively short period of time, effectively maintaining high frequency of a more virulent strain in the more sensitive host group. Our results are robust to changes in population size *N* and different proportions of *A* to *S* host individuals in the population (Figs E and F in S1 Text).

Mechanisms that lead to balancing selection and long-term coexistence can be shown to, directly or indirectly, invoke negative frequency dependence. That is, polymorphism persists if there is a mechanism favoring whichever form is rare. In this model, the increased strain diversity is maintained through indirect negative frequency dependence created by immune-heterogeneity and social stratification patterns of the host population. Each strain experiences heterogeneous selection through the opposite fitness effects that it experiences on the two different host immune-phenotypes. Here, the opposing selection pressures on the two host immuno-phenotypes generate an association between strain and host that is broken as the pathogen is transmitted to another host. Each strain escapes negative selection in an unfavorable host group through transmission to a favorable host (with probability (1 − *p*)) and increases its numbers by within-group transmission on the preferred host background (with probability *p*) and the combined effect of the two selective forces depends on the frequency of the strain in the population.

To understand the effective negative frequency dependence present in this model, we show that the rare form *V* is indeed favored in a population. Since the two strains have symmetrically opposite fitness effects on two immune phenotypes, the derivation also applies to the *v* strain when rare [57, 58]. Let us assume that the *V* strain appears on its preferred host phenotype *A* and it quickly reaches a type of “interaction-selection balance” as it is transmitted between the two host backgrounds *A* and *S*. Interaction (host switching, 1 − *p*) in this formulation mirrors mutation. At equilibrium, the frequency of *V* on host phenotype *A*, *x*, is given by

$$\begin{array}{c} \left(w_{SV}-w_{AV}\right)x^{2}-\left(w_{SV}-w_{AV}+\left(1-p\right)\left(w_{SV}+w_{AV}\right)\right)x+\left(1-p\right)w_{SV}=0. \end{array}$$ (3)

This implies that the equilibrium frequency of phenotype *V* in the *A* host group is given by *f*_*AV*_:

$$\begin{array}{c} f_{AV}=\frac{w_{AV}-w_{SV}-\left(1-p\right)\left(w_{SV}+w_{AV}\right)}{2(w_{AV}-w_{SV})}+\\ \frac{\sqrt{4w_{SV}\left(1-p\right)\left(w_{AV}-w_{SV}\right)+{\left(w_{SV}-w_{AV}+\left(1-p\right)\left(w_{AV}+w_{SV}\right)\right)}^{2}}}{2(w_{AV}-w_{SV})}. \end{array}$$ (4)

The effective selection coefficient of the *V* strain, when rare, can therefore be written as

$$\begin{array}{c} s_{V}=w_{SV}\left(1-f_{AV}\right)+w_{AV}f_{AV}. \end{array}$$ (5)

When *s*_*V*_ exceeds the effective selective coefficient of the resident strain *v*, *s*_*v*_ = (*w*_*Av*_ + *w*_*Sv*_)/2 (for equal percentages of *A* and *S* host phenotypes), the *V* mutant strain is preferred, when rare in the population. The effective selection ratio *s*_*effective*_ = *s*_*V*,*rare*_/*s*_*v*,*fixed*_ is plotted in Fig 2C and shown to increase with both *s* and *p*. Under weak selection, the probability the *V* strain increases past establishment from a single copy in the population can be approximated by twice the effective selection difference between the strains [59]. A similar argument as above reciprocally holds when the strain *v* is rare in a population fixed on the *V* strain.

### The effect of temporally changing social preference of interaction on strain coexistence

Population structure in natural host populations is not constant through time, but can dynamically change, with periods of more population mixing [51, 52, 55]. We next ask how elevated rates of polymorphism in the pathogen population change when there are periodic epochs of random and non-random host patterns of interaction. This characteristic also makes our model different from other previous models specifically examining temporally-heterogeneous selection, since changes in social patterns of interaction can act to periodically stratify the population, followed by periods of host random mixing.

**Fig 3:**
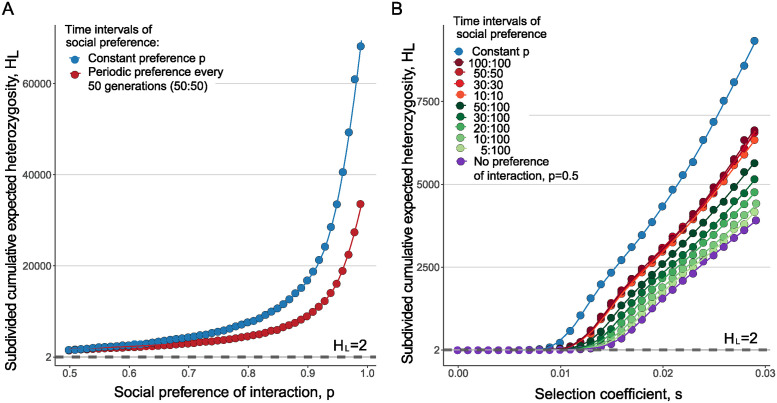
Elevated rates of polymorphism are observed even with periodic interruptions in social patterns of contact. Subdivided cumulative expected heterozygosity, *H*_*L*_ in a population of size *N* = 10^5^ and equal frequencies of *A* and *S* individuals. The dots represent ensemble averages across 10^7^ replicate Monte Carlo simulations of up to 10^7^ generations, while the lines represent cubic spline regression. (**A**) Comparison of constant preference of interaction *p* with a fluctuating through time regime, where *n*_1_ generations of preference *p* > 0.5 alternate with with *n*_2_ generations with no preference of interaction. Here, *n*_1_ = *n*_2_ = 50, and selection coefficients *s*_1_ = *s*_2_ = *s* = 0.02. (**B**) Different colors show different *n*_1_, *n*_2_ combinations, with *p* = 0.7.

We find that social preferences of interaction in the host population need not be constant for elevated pathogen heterozygosity and polymorphism to occur. We consider a periodic temporal regime, with *n*_1_ generations of interaction with fixed preference *p* > 0.5, followed by *n*_2_ generations of random host interaction, *p* = 0.5, which removes population structure. Random host interaction is expected to make intra-strain competition less concentrated in favorable hosts, thereby decreasing heterozygosity. Fig 3A shows the values of heterozygosity *H*_*L*_, with *n*_1_ = *n*_2_ = 50 over the whole range of interaction bias. The levels of strain heterozygosity are smaller when host bias changes through time, with inserted epochs of random interaction, but the temporal periods with stratified interactions are sufficient to maintain balanced patterns of pathogen polymorphism (Fig G in S1 Text).

In fact, even brief periods with non-random host pattern of interaction can promote much higher levels of pathogen coexistence than under random population mixing (Fig 3B). We explore different asymmetric combinations of *n*_1_ and *n*_2_ and show, not surprisingly, that smaller $\frac{n_{1}}{n_{2}}$ ratios lead to lower levels of balanced polymorphism. Even when bias in interaction is only briefly acting in the population (for example, social interaction preference present a tenth of the time duration), the difference in *H*_*L*_ compared to randomly mixing populations increases with increasing selective differentials between the two mutant strains.

### The effect of strain fitness differences on strain coexistence

**Fig 4:**
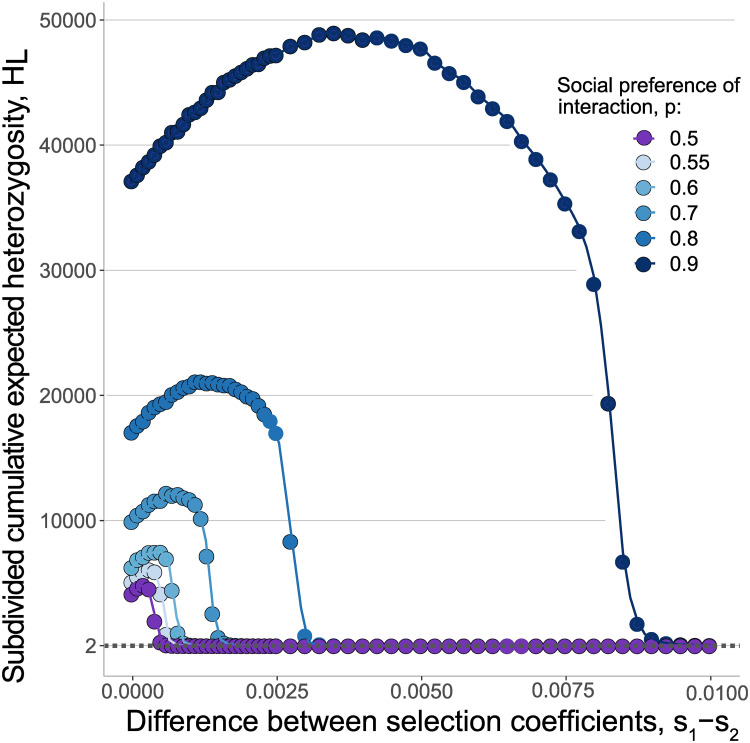
Pathogen strain polymorphism with asymmetric fitness effects between strains. Subdivided cumulative expected heterozygosity, *H*_*L*_ in a population of size *N* = 10^5^, equal frequencies of *A* and *S* individuals and selection coefficient *s*_2_ = 0.03, 0 ≤ *s*_1_ − *s*_2_ ≤ 0.01. The dots represent ensemble averages across 10^7^ replicate Monte Carlo simulations of up to 10^7^ generations, while the lines represent cubic spline regression.

Our results hold in the case of overall fitness differentials between the two strains (*s*_*d*_ = *s*_1_ − *s*_2_ > 0). With asymmetric fitness coefficients between the mutants on the two host immuno-phenotypes, the presence of social preference of interaction maintains elevated levels of strain heterozygosity and is expected to be more evident under strong social preference of interaction *p* and small fitness differentials *s*_*d*_. In Fig 4, we show a nonlinear trend in the subdivided cumulative expected heterozygosity *H*_*L*_. The strain heterozygosity increases with small differences in the selection coefficients, followed by a decrease as the difference in fitness increases. The larger the selective benefit of the wild-type strain on its preferred host type, the more pronounced this nonlinearity and the increase in population heterozygosity (Fig H in S1 Text).

### The effect of host phenotypic heterogeneity between preferentially interacting individuals

Factors such as exposure histories, age structures, and ethnicities may create immune heterogeneity within socially isolated host groups or social strata. We next explore the robustness of the balancing effect arising from our model to immune or phenotypic heterogeneity within host group (*A*/*S*). To this end, we split the population into two social subgroups, *d*_1_ and *d*_2_, each containing a mixture of both *A* and *S* individuals, and assume that hosts interact preferentially with another host in their social strata with probability *p* (regardless of host immuno-phenotype) and, with probability (1 − *p*), the interaction occurs between social subgroups (Fig A in S1 Text). We vary the proportion of *A* phenotypes in each of the two subpopulations, *P*_*d*1_(*A*) and *P*_*d*2_(*A*), and assume *P*_*d*2_(*A*) = 1 − *P*_*d*1_(*A*).

**Fig 5:**
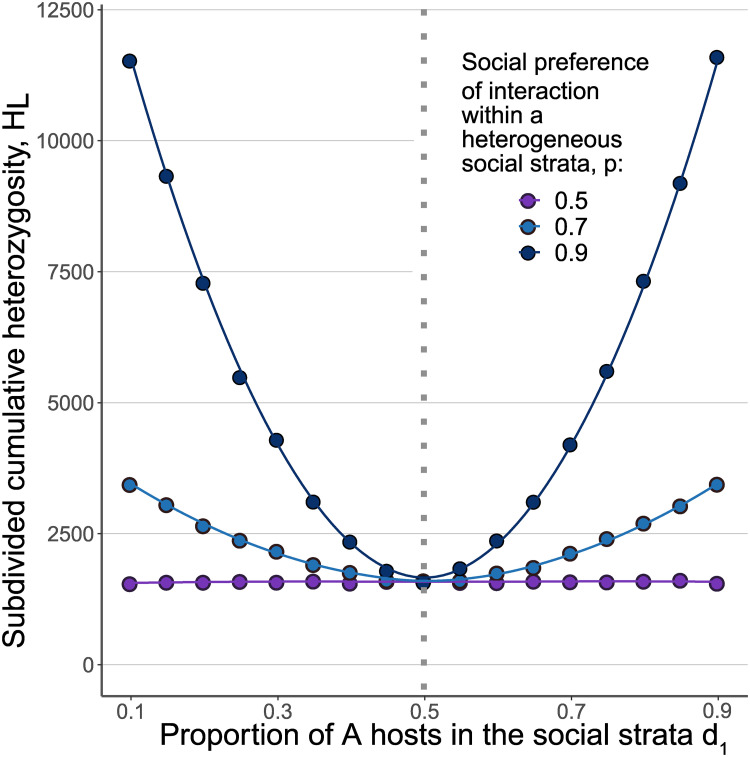
Strain coexistence with heterogeneous host population subgroups. The dots represent ensemble averages across 10^7^ replicate Monte Carlo simulations of up to 10^7^ generations, while the lines represent cubic spline regression. Here, *N*_1_ = *N*_2_ = 5 × 10^4^, *s*_1_ = *s*_2_ = 0.02 and random interaction within the subgroups but biased interaction between subgroups, as in legend. The proportion of *A* immune-phenotypes in group one is given on the *x* axis.

The strain balancing effect of heterogenous selection and social distancing is robust to within-group immune diversity and is greater with greater differences in the proportion of *A*/*S* within groups (Fig 5). However, coexistence occurs even when the two groups have the same proportions of immuno-phenotypes and are not socially distanced. As previously observed, the effect increases with *p*.

### The effect of changes in host population size and periodic host bottlenecks

Lastly, we find that the reported effect is also robust to changes in the infection rates in the host population. To accommodate for seasonal changes in infection rates, we model severe oscillating bottlenecks in the number of affected individuals and assume an oscillating population size ranging from 0.05*N* to *N* repeatedly, starting at a random point in this cycle. In Fig I in S1 Text, we show that even frequent periods of bottlenecks in the host population maintain elevated strain heterozygosity in the viral population and can promote much higher levels of pathogen coexistence than under random population mixing. Overall we find that our results are robust across a wide range of parameters and for a variety of model extensions. It is important to note that here we assume that the resident strain is already circulating in the population at the time an invader strain is introduced. While experiencing small infection rate (bottleneck) could serve as a proxy for a new outbreak, we did not model the outbreak itself and further models could accommodate for a wider set of epidemiological scenarios.

## Discussion

As populations continuously adapt from one environment to the other, genetic polymorphism provides a readily available reservoir of adaptive alleles that selection can act upon and thus promotes population persistence [60–63]. Therefore coexistence of multiple pathogen strains in a host population poses significant challenges for vaccine development or treatment outcome. For example, if a vaccine only confers immunity to a subset of viruses circulating in the population, other variants can persist and become reservoirs for viral evolution and further vaccine evasion [63–65]. A central question thus becomes: which mechanisms enable multiple strain coexistence? Here we show how social stratification, combined with the existence of distinct host immuno-phenotypes, can lead to increased levels of pathogen strain or contagious cultural behavior coexistence and highlight the importance of social preference of interaction in promoting long-lived pathogen polymorphism and rates of evolution.

Mechanisms that promote long-term coexistence and maintain balanced polymorphism in a population, can be shown to, directly or indirectly, invoke some kind of negative frequency dependence. Direct negative frequency dependence acts through the interaction of host immunity and virus immunophenotypes, whereby common strains experience more intra-host competition due to prior host exposure to similar strains [1]. This favors the evolution of antigenically diverse strains, as seen in influenza, rotavius, and HPV [66–68].

Here, we discuss a mechanism that promotes multi-strain pathogen coexistence through indirect negative frequency dependence driven by elevated biases of interaction between individuals in distinct immuno-phenotype groups in the host population. The complex interactions of human society result in heterogenous contact networks where interactions are common between some individuals in a population and are entirely absent between others [69–71]. This stratification and sparsity of connections in human populations has previously been shown to affect the culture-wide adoptions of behaviors that can have different effects on fitness in different environments or individual phenotypic backgrounds [72]. Similarly, geographic clustering of a host population, which promotes spatio-temporal structures with varying immune selection, has been shown to affect viral diversity and evolution [2, 73].

The mechanism of coexistence in our model is rooted in the fact that different strains of the pathogen experience different selection pressures when infecting hosts of different immune vulnerability, i.e. immuno-phenotypes. Since host interaction patterns can also coincide with host vulnerability, prevalence of a strain is expected to be promoted within the favorable interaction group. Because host populations are immune-heterogeneous and hosts from different groups also interact, strain prevalence is also influenced by inter-strain competitions.

We show that long-lived strain coexistence is promoted by the immune heterogeneity between hosts and the magnitude of host population stratification. Our results are robust to overall fitness differences between the two mutant strains, changes in population size *N* and different proportions of *A* to *S* host individuals in the population, as well as temporal fluctuations in interaction patterns and within-group heterogeneity. We show that temporal fluctuations in the strength of the social preference of host interaction reduce rates of polymorphism, but stratification nonetheless is the dominant effect, maintaining elevated levels of heterozygosity in the pathogen population.

It is important to note that our mechanism alone is fast enough to create multi-strain coexistence in short time scales (Fig J in S1 Text). We show that strain coexistence is achieved in a much shorter period of time in a stratified host population compared to a randomly interacting one, and it is maintained for long time scales. These results imply high evolvability in new pathogens with high transmission rates that are constrained between social groups, but allowed to spread within groups.

As a proof-of-concept, our model assumes constant proportions of *A* and *S* hosts in a population. While our robustness analysis shows that the effect we report holds across various proportions of *A* and *S* within preferentially interacting groups, we do not test temporal variation in their ratio that might arise due to immigration of hosts to the population, for example. Likewise, temporal variations in interaction preferences should consider non-deterministic patterns and, to better capture multi-strain coexistence, this model could be extended to multiple strains and multiple host groups. Further extensions of the model could also take into account other types of host-pathogen interactions. For example, different strains of a viral population can infect the same host, resulting in competition or facilitation [74, 75]. Development of acquired immunity should also be considered, especially vulnerability of re-infection [76, 77]. Additionally, as multi-strain coexistence is promoted by multiple mechanisms [1, 2, 66–68, 73], it is important to consider the interplay of these mechanisms when making evolutionary predictions.

## Supporting information

S1 Text Supporting information. This supporting information file contains the ten supplementary figures cited in the paper.
(PDF)
