# Supplementary material for: The evolutionary cost of homophily: Social stratification facilitates stable variant coexistence and increased rates of evolution in host-associated pathogens
Source: PLoS Comput Biol. 2024 Nov 22;20(11):e1012619. doi: 10.1371/journal.pcbi.1012619 (PMC11623455; doi:10.1371/journal.pcbi.1012619)
Supplement: S1 Text — This supporting information file contains the ten supplementary figures cited in the paper. (PDF) [file pcbi.1012619.s001.pdf]

Supplementary Information: Supplementary Figures

The evolutionary cost of homophily: social stratification facilitates stable variant coexistence and increased rates of evolution in host-associated pathogens

Shuanger Li[1, 3], Davorka Gulisija[2], and Oana Carja *[1]

[1]Computational Biology Department, School of Computer Science, Carnegie Mellon University, Pittsburgh, PA, USA

[2]Department of Biology, University of New Mexico, Albuquerque, NM, USA

[3]Current affiliation: Ecology and Evolution Graduate Program, University of Chicago, Chicago, USA

*To whom correspondence should be addressed. Email: oana@cmu.edu, oana.carja@gmail.com

Supplementary Figures

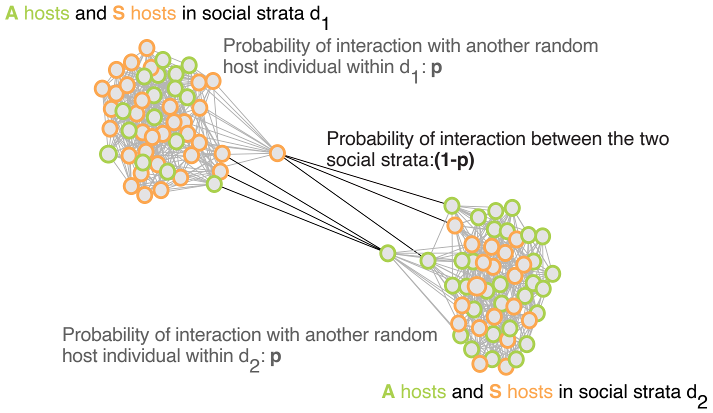

Supplementary Figure A: **Illustration of a model extension incorporating variance in immune phenotypes within host groups or social strata.** To reflect the natural demographic variance in host immuno-phenotype and test the model robustness to variance in immune phenotypes within host subgroups of preferential social interaction, we develop an extension of the model in which we model two randomly interacting population subgroups of fixed population sizes $\frac{1}{2}N$, each containing a mixture of A and S individuals.

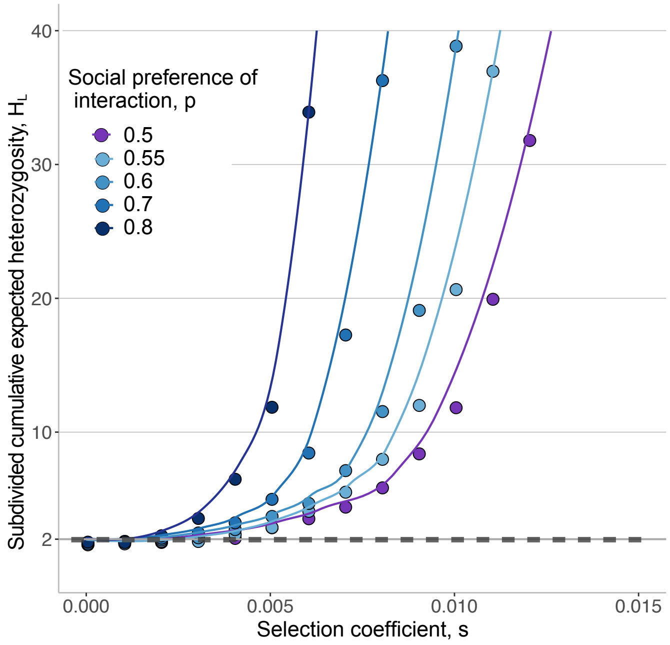

Supplementary Figure B: **Social preference of interaction promotes increased rates of strain heterozygosity and coexistence. A zoom-in view of Figure 2A for small values of p and s.** The dots represent ensemble averages across 10^7 replicate Monte Carlo simulations of up to 10^7 generations, while the lines represent cubic spline regression.

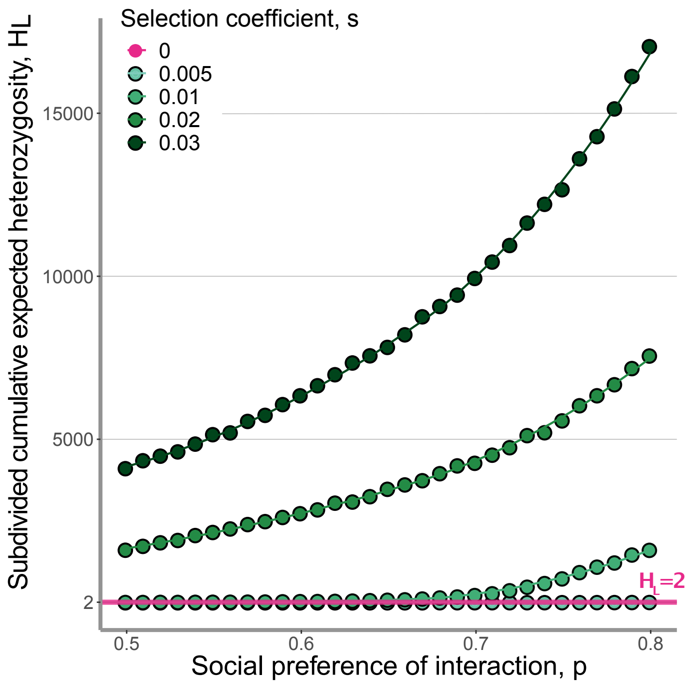

Supplementary Figure C: **Social preference of interaction promotes increased rates of strain heterozygosity and coexistence. The role of symmetric selection coefficient s.** The dots represent ensemble averages across 10^7 replicate Monte Carlo simulations of up to 10^7 generations, while the lines represent cubic spline regression. Subdivided cumulative expected heterozygosity, H_L in a population of size $N = 10^5$ with social preference of interaction p as on the x axis. The colors represent different values of selection coefficient, $s_1 = s_2 = (0, 0.005, 0.01, 0.02, 0.03)$, as presented in the legend. We show that H_L increases as p and $s_1 = s_2 = s$ increase.

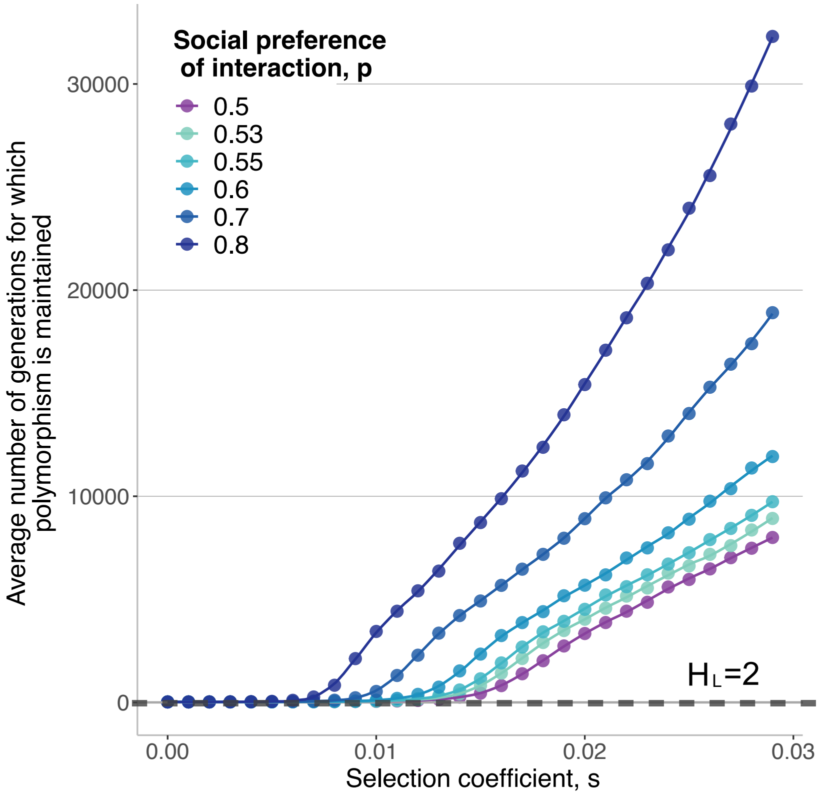

Supplementary Figure D: **Coexistence of v and V can be maintained for extended periods of time.** Average number of generations for which polymorphism is maintained as a function of the selection coefficient s. Same parameters as in **Figure 2A**.

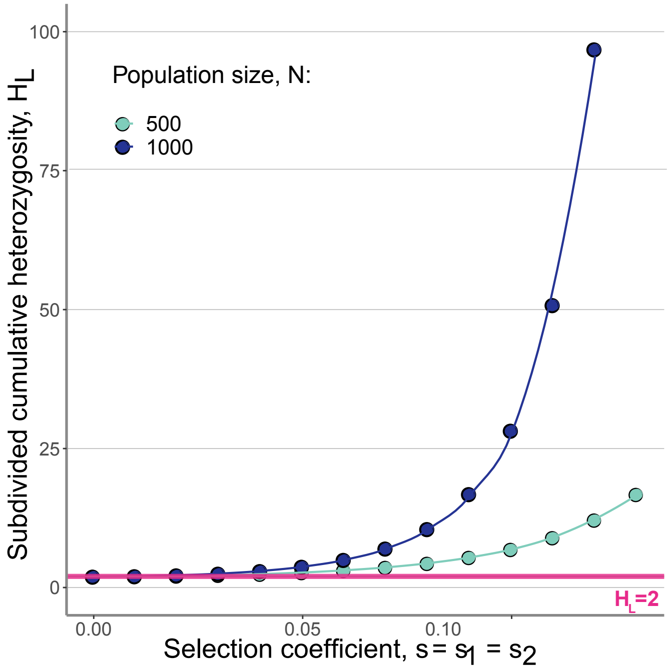

Supplementary Figure E: **Social preference of interaction promotes elevated polymorphism even when population size is small.** The dots represent ensemble averages across 10^7 replicate Monte Carlo simulations of up to 10^7 generations, while the lines represent cubic spline regression. Subdivided cumulative expected heterozygosity, H_L, in a relatively small population, with social preference of interaction $p = 0.6$. The colors represent different population sizes $N = (500, 1000)$.

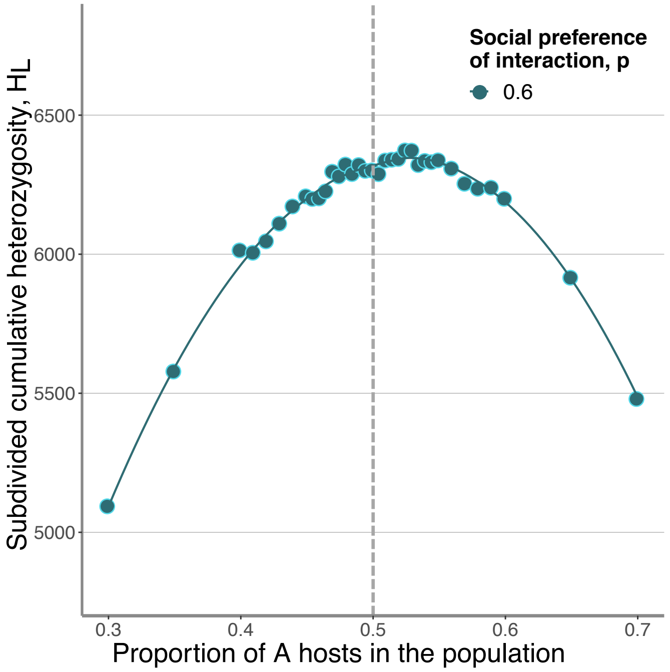

Supplementary Figure F: **Levels of heterozygosity as a function of A and S proportions in the host population**. The dots represent ensemble averages across 10^7 replicate Monte Carlo simulations of up to 10^7 generations, while the lines represent cubic spline regression. Subdivided cumulative expected heterozygosity, H_L for various frequencies of A phenotype in the host population as presented on the x axis, with $N = 10^5$, selection coefficients $s_1 = s_2 = 0.03$, and social preference of interaction $p = 0.6$.

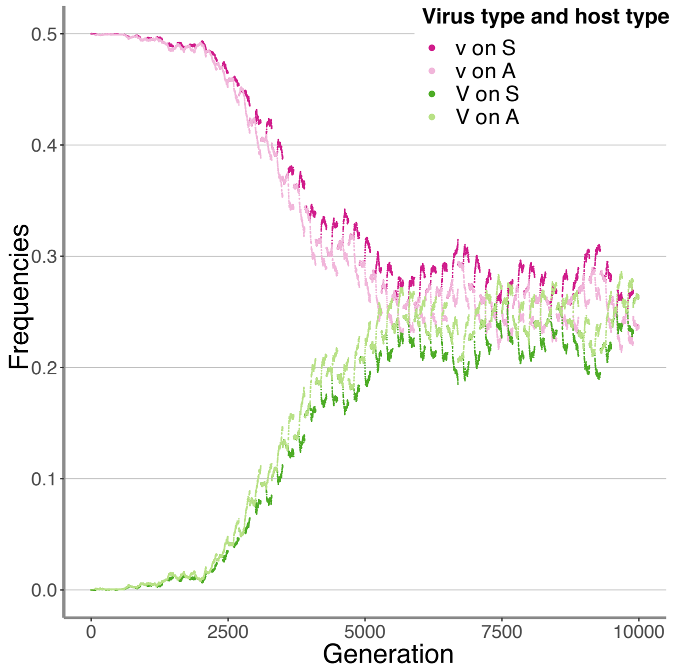

Supplementary Figure G: **Periodic presence of preference promotes pathogen strain coexistence.** Simulated population frequencies of each strain-host combination through time for one simulation run. Here, $N = 10^5$, $s_1 = s_2 = 0.02$. Periods of preference n_1 switch with periods of no preference n_2, with $n_1 = n_2 = 100$.

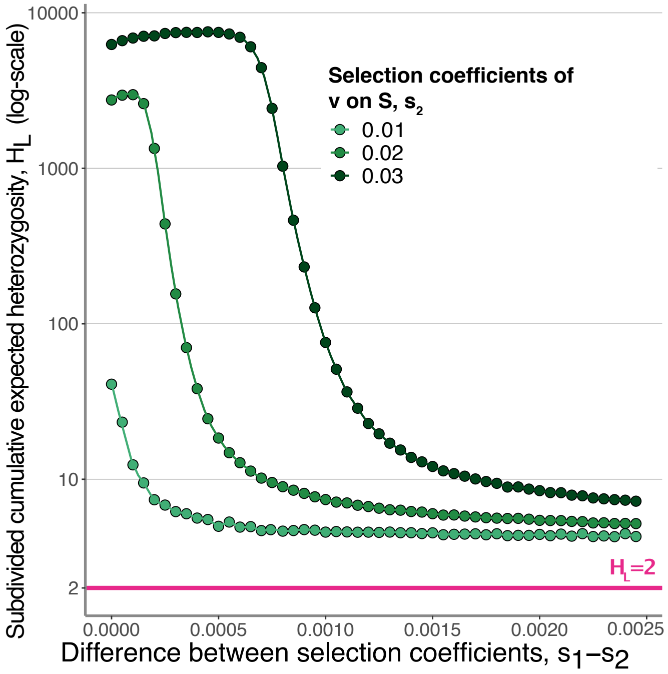

Supplementary Figure H: **Effect of selection coefficient on strain polymorphism when the two mutant strains have overall difference in fitness.** The dots represent ensemble averages across 10^7 replicate Monte Carlo simulations of up to 10^7 generations, while the lines represent cubic spline regression. Subdivided cumulative expected heterozygosity, H_L in a population of size $N = 10^5$, $0 \leq s_1 - s_2 \leq 0.0025$, and social preference of interaction $p = 0.6$. The different colors represent $s_2 = (0.01, 0.02, 0.03)$.

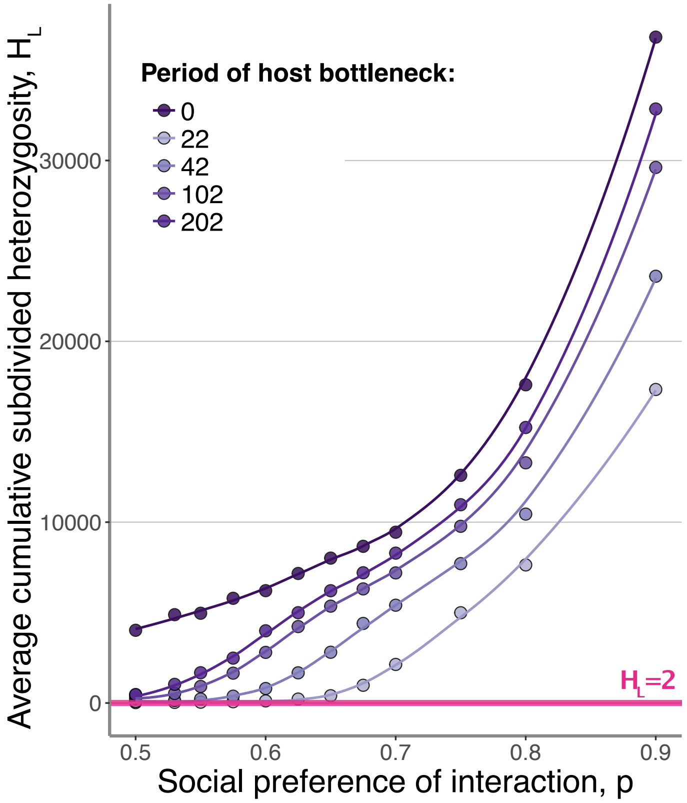

Supplementary Figure I: **Robustness of the polymorphism promoting effect by homophily to periodic changes in affected population size, including population bottlenecks.** Here, we performed $100N$ replicate simulation runs assuming an oscillating population size ranging from $0.05N$ to N repeatedly, starting at a random point in this cycle. The ratio S to A is 1, $s_1 = s_2 = 0.03$ and $N = 100000$.

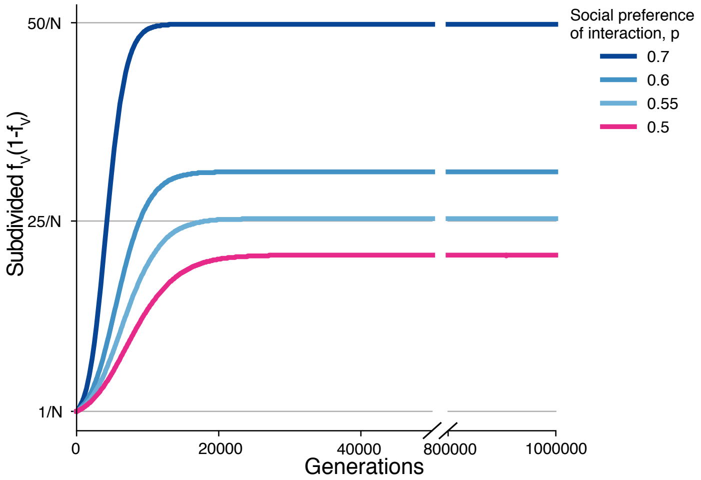

Supplementary Figure J: **Fast multi-strain coexistence in stratified host populations**. Averaged simulation results through time. Here, selection coefficient $s_1 = s_2 = 0.03$ in a population of $N = 10^5$, with 10^5 repetitions. f_V represents frequency of V on each of the host backgrounds, A and S. Subdivided $f_V \times (1 - f_V)$ measures the sum of $f_V \times (1 - f_V)$ weighted by the host population sizes: $f_{AV} \times (1 - f_{AV}) \times \frac{N_A}{N} + f_{SV} \times (1 - f_{SV}) \times \frac{N_S}{N}$.
